# Causal Mediation Analysis of Foodborne *Salmonella* Outbreaks in the United States: Serotypes and Food Vehicles

**DOI:** 10.3390/pathogens13121134

**Published:** 2024-12-22

**Authors:** Gonca Buyrukoğlu, Juan Moreira, Zeynal Topalcengiz

**Affiliations:** 1 Department of Statistics, Faculty of Science, Çankırı Karatekin University, 18100 Çankırı, Türkiye; goncabuyrukoglu@karatekin.edu.tr; 2 Department of Food Science and Human Nutrition, College of Health and Human Sciences, Colorado State University, Fort Collins, CO 80526, USA; juan.moreiracalix@colostate.edu; 3 Department of Food Science, Center for Food Safety, University of Arkansas System Division of Agriculture, Fayetteville, AR 72704, USA; 4 Department of Food Engineering, Faculty of Engineering and Architecture, Muş Alparslan University, 49250 Muş, Türkiye

**Keywords:** foodborne illnesses, pathogens, surveillance, serovar, IFSAC food categories

## Abstract

Various *Salmonella* serotypes have caused numerous foodborne outbreaks associated with food vehicles in different categories. This study provides evidence on the occurrence and inter-relations between *Salmonella* serotypes and the number of deaths mediated by the number of illnesses and hospitalizations. Confirmed foodborne outbreaks of *Salmonella* serotypes (n = 2868) that occurred between 1998 and 2021 were obtained from the Centers for Disease Control and Prevention National Outbreak Reporting System. Causal mediation analysis was performed based on 500 bootstrap samples. The serotypes and the Interagency Food Safety Analytics Collaboration (IFSAC) food categories as confounding effects were considered as categorical variables. A total of 106 single *Salmonella* serotypes were associated with foodborne outbreaks. Foodborne outbreaks caused by *Salmonella* serotypes resulted in 81,996 illnesses, 11,018 hospitalizations, and 115 deaths between 1998 and 2021 in the United States. The serotypes Enteritidis (815 outbreaks, 28.42%), Typhimurium (359 outbreaks, 12.52%), and Newport (220 outbreaks, 7.67%) accounted for almost half of *Salmonella*-linked outbreaks. Poultry products, “chickens”, “eggs”, and “turkey”, were the leading IFSAC food categories, accounting for 14.02% of total outbreaks and 10.44% of total deaths. Certain serotypes had a significant effect on illness, hospitalization, and death counts. Two serotypes, Heidelberg and Saintpaul, and “fruits” as the food vehicle in IFSAC categories had a significant direct effect on the number of illnesses, hospitalizations, and deaths as outcomes of *Salmonella* outbreaks (*p* ≤ 0.05). There was strong evidence that illness and hospitalization counts played a key role in the pathway from serotype to death counts on foodborne outbreaks caused by *Salmonella* based on causal mediation analysis. The findings of this study can help outbreak investigations and lead to prevention and control measures by providing insightful information about the frequencies of *Salmonella* serotypes and the associated food vehicles causing foodborne diseases.

## 1. Introduction

Salmonellosis represents one of the top five foodborne diseases, causing estimated annual numbers of 11% of illnesses, 35% of hospitalizations, and 28% of the deaths in the United States [1]. A wide variety of associations between *Salmonella* serotypes and attributed specific food vehicles have been known based on reported foodborne outbreaks of salmonellosis [2]. Single or multiple serotypes may play a role during a foodborne outbreak of *Salmonella*. Even though over a hundred serotypes were implicated as causative agents of human salmonellosis, some serotypes including Enteritidis, Heidelberg, Newport, and Typhimurium are predominantly reported in foodborne outbreaks [2,3,4,5,6,7]. Both typhoidal and non-typhoidal serotypes (i.e., all serotypes except Typhi, Paratyphi A, Paratyphi B, Paratyphi C, or Sendai) of *Salmonella enterica* species have the ability to cause foodborne diseases with various severities of illness resulting in hospitalization and death [8,9,10]. Certain *Salmonella* serotypes have been associated with higher numbers of foodborne outbreaks, resulting in a majority of illnesses and relative hospitalizations and deaths, than those rarely reported after investigations [9]; however, the need for comprehensive studies examining the relationships between serotypes and numbers of illnesses, hospitalizations, and deaths is emerging to understand the characterization of outbreaks.

Causal mediation analysis provides a valuable framework for understanding the complex pathways through a variable (e.g., the serotype) affecting an outcome (e.g., death counts), by examining the intermediate variables (mediators) that may lie between them. This method both assesses direct relationships and decomposes the total effect of a predictor into its direct and indirect effects, offering deeper insights into the mechanisms at play. Previously, causal mediation analysis was applied for mediating variables for substance use with challenges and recommendations about the analysis [11] and for the direct and indirect effects of motivational interviewing on dental caries count outcomes [12]. Recently, links between food and nutrition security on the perceived dietary and healthfulness of food choices were analyzed with causal mediation analysis using an observational dataset [13]. Causal mediation analysis has been widely applied for clinical research and public health subjects [14,15]. The potential use of causal mediation analysis deserves attention to identify reasons for the consequences of foodborne outbreaks.

Foodborne disease outbreak surveillance involves the identification of implicated foods, pathogens as etiological agents, food preparation and consumption settings, points of contamination, and changes in outbreak trends over time [5]. In the United States, available data regarding investigated foodborne outbreaks by all states are reported by the Centers for Disease Control and Prevention through the National Outbreak Reporting System (NORS) [16]. Foodborne disease outbreak surveillance by NORS provides the numbers of illnesses, hospitalizations, and deaths for each outbreak attributed to food vehicles in the Interagency Food Safety Analytics Collaboration (IFSAC) food categories. During the last decade, several studies have compiled the descriptive foodborne outbreak statistics of available data for specific year intervals, pathogens, and food vehicles and categories to help the improvement of public health in the United States [3,4,6,7,9,17,18,19]. In this study, foodborne outbreaks of *Salmonella* between 1998 and 2021 in the United States were extracted from the NORS database to understand trends in *Salmonella* serotypes and implicated IFSAC food categories. The occurrence and inter-relations between *Salmonella* serotypes and the number of deaths mediated by the numbers of illnesses and hospitalizations were investigated through causal mediation analysis.

## 2. Materials and Methods

### 2.1. Dataset

The dataset of foodborne outbreaks (n = 2868) linked to laboratory-confirmed etiologies of *Salmonella* serotypes was extracted from the Centers for Disease Control (CDC) National Outbreak Reporting System (NORS) for the period from 1998 to 2021 in the present study with a last transfer date of 17 April 2023 (https://wwwn.cdc.gov/norsdashboard/). Outbreaks of *Salmonella* included all typhoidal and non-typhoidal single serotypes causing at least 10 outbreaks (n = 36), multiple serotypes, unknown serotypes, and a total of “other” serotypes (n = 73) listed as causative etiological agents a maximum of nine times by NORS. Incomplete data in the number of death counts (n = 19) were accepted as 0 for the analysis.

The same criteria of confirmed food vehicles implicated in at least ten *Salmonella* outbreaks (n = 16) were applied for IFSAC food categories including “chicken”, “eggs”, “pork”, “fruits”, “beef”, “turkey”, “seeded vegetables”, “dairy”, “sprouts”, “nuts-seeds”, “vegetable row crops”, “fish”, “crustaceans”, “herbs”, “other meat”, and “root/underground”. “Uncategorized” (n = 1308) and “multiple” (n = 617) food categories were separated from the other (n = 29) implicated foods by pooling all remaining categories of “other” (n = 9), “grains-beans” (n = 8), “other poultry” (n = 2), “game” (n = 2), “aquatic animals” (n = 1), “mollusks” (n = 4), “fungi” (n = 2), and “oils-sugars” (n = 1).

### 2.2. Causal Mediation Analysis with Two Mediators

The regression-based approach for causal mediation analysis proposed by Valeri et al. [20] and VanderWeele et al. [21] was applied in the present study as described below. For a count outcome, causal effects were estimated on the ratio scale (Table 1). All causal effects were estimated through direct counterfactual imputation estimation. Standard errors of the causal effects were estimated through bootstrapping. An intermediate variable followed the causal pathway from exposure to outcome, and a confounding variable was defined as a variable resulting in an outcome. The confounding variable differed from the intermediate variable but was associated with the factor under investigation.

#### 2.2.1. Assignment of Mediators

**Figure 1:**
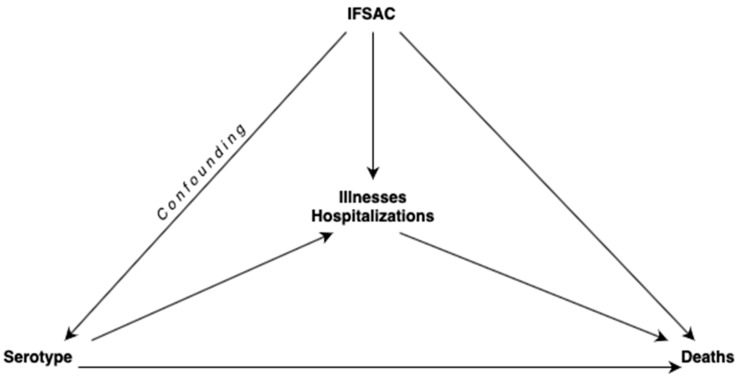
Mediation with illnesses and hospitalizations (two mediators, $M=\left(M^{\left(1\right)},\;M^{\left(2\right)}\right)$; serotype (exposure A); confounder, not affected by IFSAC (exposure C); and deaths (outcome Y).

The causal mediation analysis with illnesses and hospitalizations as two mediators (*M* = $\left(M^{\left(1\right)},\;M^{\left(2\right)}\right)$) included the serotype (denoted as exposure A); the confounder, not affected by IFSAC food categories as confounding variables that may affect the exposure (denoted as exposure C); and deaths (outcome denoted as Y). The relationships among exposures, mediators, and outcome are given in Figure 1.

When assuming that there were multiple mediators of interest, $M=\left(M^{\left(1\right)},\;M^{\left(2\right)}\right)$, and that we were interested in the effects mediated through $M=\left(M^{\left(1\right)},\;M^{\left(2\right)}\right)$ jointly and independent of mediators, the controlled direct effects, natural direct effects, and natural indirect effects could be estimated under the following assumptions [22]:

“There are no unmeasured exposure-outcome confounders given C”.

“There are no unmeasured mediator-outcome confounders given (C, A)”.

“There are no unmeasured exposure-mediator confounders given C”.

“There are no mediator-outcome confounders affected by exposure”.

If the exposure was categorical, the outcome and the mediators were counted. When the confounder was categorical, estimates of the causal effects could be calculated via the generalized linear models. The following three separate regression models were utilized: one for the outcome Y on the exposure A, mediators and confounding variable C, a second and a third regressions for $M^{\left(1\right)}$ and $M^{\left(2\right)}$ on the exposure A, and confounding variable C. Those regressions were combined to estimate natural direct and indirect effects.

#### 2.2.2. Negative Binomial Regression Model

Count data are usually modeled with the Poisson regression model. The common assumption for Poisson regression is equality of variance and mean. However, this assumption is often violated in practice. When the overdispersion exists, negative binomial (NB) regression should be considered. This model had a built-in dispersion parameter so that it accommodated at greater variance than the mean [23]. Those NB regressions were combined under the generalized linear model (glm) fit to estimate natural direct and indirect effects (Tables S1–S3).

#### 2.2.3. Mediation Analysis

The mediation models were specified with the number of illnesses ($M^{\left(1\right)}$) and the number of hospitalizations ($M^{\left(2\right)}$) as mediating the association between the serotype and the number of deaths (Y). The model was performed on serotypes (exposure variable) and IFSAC food categories (confounder) as categorical variables. The inference method for estimating the standard errors of causal effects was bootstrap, and the model was based on 500 bootstrap samples. Serotype categories with the highest risk of death were examined. The effect of the serotypes (exposure) on death was mediated by illnesses and hospitalizations. Negative binomial regression models were implemented for the outcome (Y) and the mediators considering the count nature of the data. The model included categorical variables for both serotypes and IFSAC food categories.

The glm model fitted for the first mediator was as follows:

(1) $$g\left(E\left[M^{\left(1\right)}|A,C\right]\right)={\beta_{0}}^{\left(1\right)}+\sum_{h=1}^{H}{\beta_{1h}}^{\left(1\right)}I\left\{A=h\right\}+\sum_{t=1}^{T}{\beta_{2t}}^{{\left(1\right)}^{\prime }}I\left\{C=t\right\}+\varepsilon_{M^{\left(1\right)}}$$

The glm model fitted for the second mediator was as follows:

(2) $$g\left(E\left[M^{\left(2\right)}|A,C\right]\right)={\beta_{0}}^{\left(2\right)}+\sum_{h=1}^{H}{\beta_{1h}}^{\left(2\right)}I\left\{A=h\right\}+\sum_{t=1}^{T}{\beta_{2t}}^{{\left(2\right)}^{\prime }}I\left\{C=t\right\}+\varepsilon_{M^{\left(2\right)}}$$

The glm model fitted for the outcome, $\theta_{1h}$, was as follows:

(3) $$\begin{array}{l} g\left(E\left[Y|M^{\left(1\right)},M^{\left(2\right)},A,C\right]\right)\\ \;\;\;\;\;=\theta_{0}+\sum_{h=1}^{H}\theta_{1h}I\left\{A=h\right\}+{\theta_{2}}^{\left(1\right)}M^{\left(1\right)}+{\theta_{2}}^{\left(2\right)}M^{\left(2\right)}\\ \;\;\;\;\;+\sum_{t=1}^{T}{\theta_{4t}}^{\prime }I\left\{C=t\right\}+\varepsilon_{Y} \end{array}$$

where *Y* denotes outcome of interest for each individual; *A* is the exposure; C is a set of covariates (confounder); M denotes the intermediate variables (on the pathway between *A* and *Y*); β and θ are the corresponding regression coefficients; and $\varepsilon_{M^{\left(1\right)}}$, $\varepsilon_{M^{\left(2\right)}}$, and $\varepsilon_{Y}$ are independent random errors. The regressions in Equations (1)–(3) can be combined to estimate causal effects as shown in Table 1.

where a and $a^{*}$ are the active and the reference values for exposure (A), and m is the value at which the mediators are controlled. $M_{a}$ represents the counterfactual outcome mediators that could have been seen had the exposure been set to be a. $Y_{am}$ indicates the counterfactual value of the outcome that could have been seen had exposure been set to be a and the mediator to be m.
  (4) $$R^{CDE}=exp\left\{\sum_{h=1}^{H}\theta_{1h}I\left\{a=h\right\}-\sum_{h=1}^{H}\theta_{1h}I\left\{a^{*}=h\right\}\right\}$$

(5) $$\begin{array}{l} R^{PNIE}=exp\{{\theta_{2}}^{\left(1\right)}\left(exp\left\{\sum_{h=1}^{H}{\beta_{1h}}^{\left(1\right)}I\left\{a=h\right\}-\sum_{h=1}^{H}{\beta_{1h}}^{\left(1\right)}I\left\{a^{*}=h\right\}\right\}\right)\\ \;\;\;\;\;\;+{\theta_{2}}^{\left(2\right)}\left(exp\left\{\sum_{h=1}^{H}{\beta_{1h}}^{\left(2\right)}I\left\{a=h\right\}-\sum_{h=1}^{H}{\beta_{1h}}^{\left(2\right)}I\left\{a^{*}=h\right\}\right\}\right)\} \end{array}$$

As the exposure–mediator interaction was not taken into account for this study, $R^{CDE}=R^{PNDE}=R^{TNDE}$ and $R^{PNIE}=R^{TNIE}$.

The analysis was performed using the MASS [24] and CMAverse [25] packages in RStudio programming language version 2023.12.11 [26]. The following significance codes were accepted for statistical comparison of the calculated parameters within the dispersion and negative binomial regression model: 0, ***; 0.001, **; 0.01, *; 0.05, ‘.’. 

## 3. Results

### 3.1. Frequency of Serotypes Linked to Salmonella Outbreaks

The frequency of foodborne outbreaks in the United States associated with *Salmonella* serotypes between 1998 and 2021 listed by the NORS database with confirmed pathogens is shown with the resulting numbers of illnesses, hospitalizations, and deaths in Table 2. A total of 106 single serotypes were associated with foodborne outbreaks. *Salmonella* serotypes were associated with a total of 2868 laboratory-confirmed foodborne outbreaks resulting in 81,996 illnesses, 11,018 hospitalizations, and 115 deaths from 1998 to 2021 in the United States. The serotypes Enteritidis (815 outbreaks, 28.42%), Typhimurium (359 outbreaks, 12.52%), and Newport (220 outbreaks, 7.67%) accounted for almost half of *Salmonella*-associated outbreaks. Relatively, these serotypes also caused the highest numbers of illnesses (46.1%), hospitalizations (40.58%), and deaths (45.21%) in total. The serotypes Heidelberg (165 outbreaks, 5.75%) and Javiana (95 outbreaks, 3.31%) were ranked fourth and fifth, causing foodborne outbreaks as single causative agents. There were outbreaks of *Salmonella* serotypes resulting in unproportional numbers of illnesses, hospitalizations, and deaths. The serotype Saintpaul caused 1.85% (53 outbreaks) of *Salmonella* outbreaks, where the percentages of illnesses (3.76%) and hospitalizations (5.59%) were calculated two to three times more compared with the percentage of outbreaks caused by the same serotype. Similarly, the serotype Poona caused 0.35% (10 outbreaks) of outbreaks, resulting in 1.24% of total illnesses, 2.21% of total hospitalizations, and 6.96% of total deaths by all outbreaks of *Salmonella* serotypes.

### 3.2. Frequency of IFSAC Food Categories Linked to Salmonella Outbreaks

The frequency of IFSAC food categories linked to *Salmonella* outbreaks in the United States between 1998 and 2021 listed by the NORS database with confirmed pathogens is shown with the resulting number of illnesses, hospitalizations, and deaths in Table 3. Almost half of food vehicles causing *Salmonella* outbreaks (1308 outbreaks, 45.61%) were not placed in any IFSAC food categories. “Chicken” (177 outbreaks, 6.17%), “eggs” (160 outbreaks, 5.58%), and “pork” (99 outbreaks, 3.45%) were reported as the top three food categories implicated in *Salmonella*-linked outbreaks. There were outbreaks of *Salmonella* resulting in higher percentages of illnesses, hospitalizations, and deaths than those of outbreaks associated with a single food vehicle. Even though food vehicles in the categories of “fruits” (83 outbreaks, 2.89%), “seeded vegetables” (59 outbreaks, 2.06), and “nuts-seeds” (22 outbreaks, 0.77%) were implicated in around 5% of *Salmonella* outbreaks in total, all these outbreaks resulted in 15.04% of all illnesses, 20.49% of all hospitalizations, and 36.52% of all deaths.

### 3.3. Data Summary for Frequency of Serotypes Linked to Salmonella Outbreaks

The frequencies (as percentages) of illness, hospitalization, and death counts associated with *Salmonella* outbreaks are shown in Table 4. Outbreak numbers of *Salmonella* serotypes causing fewer than ten hospitalizations and deaths accounted for 92.33% and 100% of all 2868 analyzed outbreaks (as shown in Table 2), respectively. Illness counts with a number of cases below 100 represented 95.02% of all reported outbreaks. Table 5 shows the magnitude of the calculated dispersion parameters and corresponding *p*-values for each response variable. A considerably large degree of overdispersion pertained to illness counts (134.5764) (*p* < 0.000). The smallest yet statistically significant magnitude of overdispersion belonged to death counts (1.2177) (*p* < 0.05).

### 3.4. Illness, Hospitalization, and Death Counts by the Negative Binomial Regression Model

Table 6 summarizes NB regression results for the illness counts ($M^{\left(1\right)}$), the hospitalization counts ($M^{\left(1\right)}$), and the death counts (Y) due to outbreaks of *Salmonella* serotypes with statistically significant model parameters for all three models. For the detailed model summary, please see Tables S1–S3 in the Supplementary Materials. Five single *Salmonella* serotypes (Braenderup, Heidelberg, Javiana, Montevideo, and Saintpaul) and thirteen IFSAC food categories as the food vehicle (“beef”, “chicken”, “dairy”, “eggs”, “fish”, “herbs”, “nuts-seeds”, “pork”, “root/underground”, “seeded vegetables”, “sprouts”, “turkey”, and “vegetable row crops”) resulted in a significant effect on illnesses caused by *Salmonella* outbreaks (*p* < 0.05). Nine *Salmonella* serotypes (Heidelberg, I 4,[5],12:i:-, Javiana, Newport, Oranienburg, Poona, Reading, Saintpaul, and Typhimurium) and eleven IFSAC food categories (“beef”, “chicken”, “dairy”, “fruits”, “herbs”, “nuts-seeds”, “pork”, “root/underground”, “seeded vegetables”, “sprouts”, and “turkey”) provided a significant effect on hospitalizations (*p* < 0.05). Three serotypes, Baildon, Heildelberg, and Saintpaul, and “fruits” as the only food vehicle in IFSAC food categories were found to be statistically significant in relationship to the number of deaths caused by outbreaks of *Salmonella* serotypes (*p* < 0.05).

### 3.5. Results of Causal Role of Mediators

**Figure 2:**
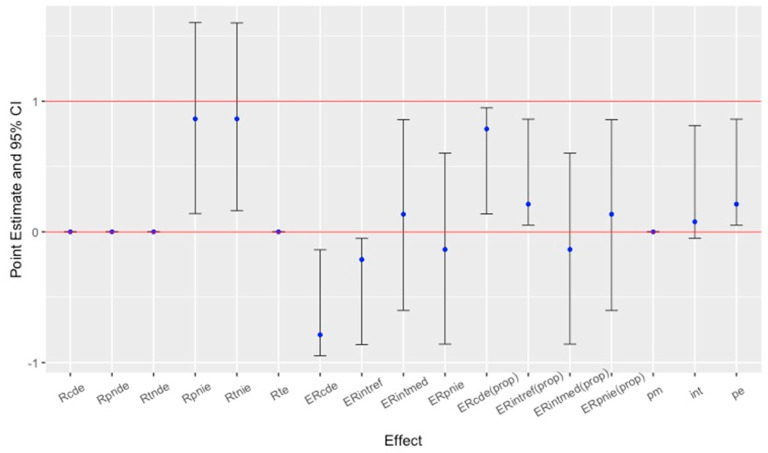
The results of causal mediation analysis with hospitalization and illness counts as mediators for deaths related to foodborne outbreaks caused by *Salmonella* serotypes. (Rcde, controlled direct effect rate ratio; Rpnde, pure natural direct effect rate ratio; Rtnde, total natural direct effect rate ratio; Rpnie, pure natural indirect effect rate ratio; Rtnie, total natural indirect effect rate ratio; Rte, total effect rate ratio; ERcde, excess relative rate due to controlled direct effect; ERintref, excess relative rate due to reference interaction; ERintmed, excess relative rate due to mediated interaction; ERpnie, excess relative rate due to pure natural indirect effect; ERcde(prop), proportion of ERcde; ERintref(prop), proportion of ERintref; ERintmed(prop), proportion of ERintmed; ERpnie(prop), proportion of ERpnie; pm, overall proportion mediated; int, overall proportion attributable to interaction; pe, overall proportion eliminated.)

In causal mediation analysis, the indirect, direct, and total effect rate ratios (in Table 1) were calculated based on the regression parameter estimates in Tables S1–S3 more broadly of each pathway (through the two count mediators and directly from the serotype to the outcome). Figure 2 presents the point estimate and 95% confidence intervals of causal mediated effect rates of illnesses (first mediator) and hospitalizations (second mediator) to death counts caused by outbreaks of *Salmonella*. These result of causal mediation analysis with hospitalization and illness counts can be found in Table S4 in detail. An $R^{CDE}$ of 0 indicated that when the illness and hospitalization counts were held constant at a specified level, serotypes of *Salmonella* had no direct effect on the death counts. In practical terms, an $R^{CDE}$ of 0 suggested that the death count would be reduced to zero in the presence of the serotype as a variable, independent of the mediators. A rate ratio less than 1 suggested a reduction in the death counts. An $R^{PNIE}$ of 0.865 indicated that when holding all other factors at a constant level, the serotype was associated with an expected 13.5% decrease in the death count through the indirect pathway involving the mediators. The standard error of 47.366 suggested a high level of variability in the estimate, implying a substantial variability in the data.

The calculated *p*-value indicated that the indirect effect was not statistically significant (*p* = 0.216 > 0.05). While the estimated indirect effect suggested a decrease in the outcome via the mediators, this effect was not statistically strong enough to rule out due to random chance. A total natural indirect effect rate ratio ($R^{TNIE}$) of 0.865 implied that the effect of serotype on death count operating through both mediators was associated with a decrease in the rate of death by 13.5% with an indication of a protective indirect effect via these mediators in relation to the death count (outcome). Since the total effect rate ratio was the product of a direct effect and an indirect effect, it was calculated as 0 ($R^{TE}=0$). Negative excess relative rates indicated a protective effect of the serotype on the death rate. This might have mitigated some of the adverse effects of the mediators on the death count by serotype variability. Specifically, an ${ER}^{CDE}$ of −0.788 indicated a 78.8% decrease in the rate of the death count due to the controlled direct effect of the serotype, independent of the pathways through illness and hospitalization counts. After accounting for the influence of illness and hospitalization counts, serotypes were associated with a substantial reduction in death counts.

The overall proportion eliminated was found to be 0.212 (CI—0.05, 0.863, *p*-value = 0.008, ≤0.05), suggesting that 21.2% of the total effect of the serotype on death count operated through the mediators. Here, there was strong evidence that illness and hospitalization counts played a key role in the pathway from serotype to death count in foodborne outbreaks caused by *Salmonella*. It implied that controlling or modifying the mediators could potentially lead to a meaningful reduction (21.2%) in the effect of the serotype categories on death counts.

## 4. Discussion

The availability of surveillance information of foodborne disease outbreaks provided by national reporting systems and public health departments helps researchers to examine the number of people affected by foodborne outbreaks. In most cases, the frequencies of foodborne outbreaks have been extracted to understand trends in reported illnesses, hospitalizations, and deaths with associated pathogens and implicated foods for a period of years. Missing or incomplete data, the presence of suspected or confirmed etiology in the dataset, and unknown food categories limit the analysis of relationships between pathogens and food vehicle causing foodborne outbreaks [5]. In the present study, only confirmed outbreaks of *Salmonella* reported from 1998 to 2021 by NORS were extracted to understand causal relationships between serotypes and death counts mediated by the numbers of illnesses and hospitalizations in addition to the summary of foodborne outbreak frequencies associated with serotypes linked to food categories in the United States.

Several studies have been published about the frequencies of foodborne outbreaks for all or specific causative pathogens and food groups within various periods in the United States as mentioned in the Introduction Section. Overall, *Salmonella* spp. have been reported as some of the most concerning pathogens of outbreaks associated with diverse types of food vehicles. The reported numbers of illnesses, hospitalizations, and deaths linked to outbreaks of foodborne pathogens in the literature may have some variabilities due to limitations such as missing or incomplete data, investigated but not reported data, the presence of suspected or confirmed etiology in the dataset, and unknown food vehicles and pathogens [5,9,19]. Also, surveillance reporting systems such as NORS may have variations in released information depth of reporting outbreak data across the states depending on the availability of state and local resources with the possibility of later updates and deletions in the reporting system [9,19]. However, frequencies in foodborne outbreaks are comparable in most cases to understanding general trends in causative pathogens and implicated food vehicles.

In the present study, the most predominant *Salmonella* serotypes associated with foodborne outbreaks were determined as Enteritidis (28.42%), Typhimurium (12.52%), and Newport (7.67) from 1998 to 2021 (Table 2). Similarly, Enteritidis (29.1%), Typhimurium (12.6%), and Newport (7.6%) also accounted for nearly half of all *Salmonella* outbreaks in the previously evaluated period between 1998 and 2015 [7]. This trend indicates that these three serotypes continue to be predominant and virulent despite the emergence of other serotypes and changes in food vehicles commonly associated with this pathogen. The same predominant serotypes of *Salmonella*, Enteritidis and Typhimurium, are also a major concern of foodborne outbreaks in Europe [27]. Despite the high frequency of these three serotypes of *Salmonella* associated with foodborne outbreaks, the serotypes Braenderup, Heidelberg, Javiana, Montevideo, and Saintpaul are determined significantly in relationship to number of illnesses (*p* < 0.05) (Table 6). As a result, various strains of mentioned serotypes are commonly used in the persistence and challenge studies because of their accepted growth and survival abilities in the food products associated with *Salmonella* outbreaks.

Apart from virulence, the serotype Typhimurium has consistently maintained elevated mortality rates (17.39% of total deaths are attributed to Typhimurium). One reason for this serotype maintaining high mortality rates may include multi-drug-resistant (MDR) strains that can complicate medical treatments [28]. The MDR *Salmonella* outbreaks have commonly been associated with animal-source food products, raising concerns of antimicrobial plasmid transferal in microbial populations [3,29,30,31,32]. Outbreaks involving MDR strains have been found to be 82% related to land animals, and even more concerning, of those outbreaks resistant to quinolones, 89% were related to land animals. This makes the elevated mortality rates a two-fold problem, in which enhanced virulence drives up illnesses and hospitalizations, and resistance to the main treatment for salmonellosis (quinolones) complicates the treatment of these patients further, leading to high mortality rates. The three serotypes holding the bulk of resistant strains of outbreaks include Heidelberg, Newport, and Typhimurium [33]. In the present study, the same serotypes had a significant effect on hospitalization counts, supporting previously reported serotypes causing an elevated risk for public health (in addition to the serotypes I 4,[5],12:i:-, Javiana, Oranienburg, Poona, Reading, and Saintpaul) (*p* ≤ 0.05) (Table 6).

The disproportionate numbers of illnesses and hospitalizations from outbreaks involving the serotype Saintpaul indicate knowledge gaps related to how this serotype affects patients. The results of the present study also show that the serotypes Baildon and Saintpaul significantly affect the number of death counts (*p* ≤ 0.05) (Table 6). This relatively uncommon serotype compared with Enteritidis, Typhimurium, and Newport has exposed limitations in outbreak investigations related to it. In 2008, there was a multi-state outbreak of *S.* Saintpaul in which confusion regarding the food vehicle led to mixed public messaging and contributed to this single outbreak having 1500 associated illnesses [34,35]. Challenges in detecting the serotype Saintpaul and narrowing down food vehicles may be one factor that leads to increased illnesses and hospitalizations. However, this serotype’s increased virulence may be a more influential driving factor. The serotype Saintpaul has been found to carry virulence genes associated with the prophages Gifsy-1 and Gifsy-2, causing delayed or reduced immune system response in human patients [36]. The frequent association of Saintpaul with fruits and vegetables is a possible indication for this serotype’s increased risk of leading to illness or hospitalization [37,38].

Despite only causing 10 foodborne outbreaks during the evaluated period, *S.* Poona was responsible for 6.96% of total deaths. More than 50% of outbreaks involving *S.* Poona have been associated with fruits and vegetables [2]. A single outbreak in 2015–2016 involving cucumbers accounted for 907 illnesses and led to a recall from 40 states [39]. In addition to being predominantly in food vehicles with a lack of pathogen control methods, the serotype Poona has also been related to low-water-activity foods such as a rice-based infant formula outbreak that included three European countries [40]. Increased virulence compared with other serotypes and possible thermal resistance are possible explanations for Poona’s elevated illness, hospitalization, and death counts despite accounting for few outbreaks [41]. 

Poultry-related products (“chickens”, “eggs”, and “turkey” as IFSAC food categories) accounted for 14.02% of total *Salmonella* outbreaks and resulted in 10.44% of total deaths. Foods in this category have consistently been responsible for large portions of *Salmonella* outbreaks with a significant effect on the numbers of illnesses and hospitalizations (*p* ≤ 0.05) in the United States despite efforts to monitor and reduce these contaminations (Table 6). Basler et al. [42] attributed this latency in case reduction to a shift in practices regarding poultry, particularly backyard poultry flocks in homes and contact with children. This is a main concern with children as several *Salmonella* outbreaks have been caused by contact with animals instead of contaminated food vehicles. Of outbreaks involving children from 1 to 4 years of age, 5.6% were related to the consumption of food, and 24.5% were related to contact with animals [6]. Egg-associated outbreaks are also affected by increased production in the last decades [43]. Despite the availability of eggs treated for pathogen reduction such as pasteurized eggs, consumer practices are a driving factor in the propagation of *Salmonella*, and improper cooking temperatures and post-cooking holding conditions are leading to the growth of this pathogen [44]. It seems that poultry products continue to be a concern for *Salmonella* worldwide due to increases in associated serotypes, failures in production systems, and unsafe consumer practices [45].

In the present study, “fruits” as an IFSAC food category represented the highest amount of *Salmonella* outbreaks that were not associated with an animal source (“chickens”, “eggs”, and “pork”). Also, “fruits” were determined as the only food category with a significant effect on the number of deaths caused by *Salmonella* serotypes (*p* ≤ 0.05) (Table 6). Agricultural commodities such as “fruits”, “seeded vegetables”, and “nuts-seeds” accounted for over one-third of the total deaths from *Salmonella* outbreaks (36.52%). As indicated by Hanning et al. [46], *Salmonella* outbreaks are found to be increasingly caused by the interaction of animals that may carry *Salmonella* and their feces with agricultural water sources and produce fields. Elongated persistence of *Salmonella* strains in agricultural water sources and domesticated and wild animal feces increases the chance of contamination in the produce fields [47,48,49,50]. This epidemiological shift of more frequent *Salmonella* outbreaks related to fruits and vegetables is not only an isolated phenomenon in the United States. Australia has also seen an increased amount of *Salmonella* outbreaks related to fruits and vegetables, with 4–8% of the total *Salmonella* outbreaks being associated with these commodities [51]. This trend seems to be linked to production systems, however the emergence of new *Salmonella* serotypes such as Agona, Anatum, Oslo, Poona, and Saintpaul are also a concern as these were rarely associated with outbreaks before and are now commonly found in fruit- and vegetable-related outbreaks [51]. Global market changes surrounding fruits and vegetables can also be considered to have a significant impact in driving *Salmonella* outbreaks with an increasing distribution of these products. The increased availability of ready-to-eat products is helping the spread of *Salmonella* around the country, with most multistate outbreaks related to produce as a food vehicle [52,53]. Mass distribution and shorter shelf lives can lead to batch contamination and multi-state outbreaks due to the expanse of modern distribution networks.

The 2.82% of *Salmonella* outbreaks related to “beef” as a food vehicle is in a sharp decline compared with the 4.89% reported from 1973 to 2011 [54]. Additionally, *Salmonella* outbreaks related to “beef” have been reported to be disproportionately related to ground beef (45%) [54]. For example, ground beef accounted for 44% of *Salmonella* outbreaks associated with meat, and these outbreaks had a high public health impact with 73% of illnesses from this specific category [3]. The spread of outbreaks related to “beef” is accelerated by 96.7% of illnesses involving highly virulent serotypes [55]. Beef cattle production methods and ground beef providing an ideal growth environment for *Salmonella* are markedly the main reasons why “beef” remains a predominant food vehicle for *Salmonella* outbreaks.

Negative binomial regression models were used due to the overdispersion in the dataset. If the overdispersion is ignored, the models’ performances are affected negatively. Overdispersion can cause underestimated or deflated standard errors of the parameter estimates. That is, researchers may decide a variable to be a significant predictor, while it is actually not. Thus, overdispersion is needed to be carefully taken into account in the models [56]. This study highlights the implementation of a solution for the investigation complexities of mediation analysis for overdispersed count outcomes. The two-stage framework used for the NB model and mediation analysis (Figure 1) allowed us to decompose the natural direct effect under the aforementioned assumptions. The main benefit of this study is to strengthen the applied researchers’ tool kit along with providing quantitative methodologists as a novel direction for exploring the indirect associations with the expanding nonlinearity in mediation model. The effect of serotypes on death would have been higher, in fact, than if the causality were ignored.

There were two main limitations in this study. The first limitation was that the mediators could be sequential. Incorporation of this sequentiality ($A\to M^{\left(1\right)}\to M^{\left(2\right)}\to Y$) can be considered in future research. The calculation of conditional mediated effects would be more challenging for models with sequential mediators since the mediated effect includes more estimates with each added mediator in these models. The second limitation is that there are considerable large numbers of zero counts in the outcomes. Although the zero-inflated and hurdle models were implemented [57,58], these models never converged. The reason may be that the determinant of the hessian matrix in the deep theory is somehow zero or close to zero. One alternative way could be to perform transformation of the variables and make these models fit. One remarkable point regarding this study is consideration of the causality between the mediators and outcome. When causality is ignored due to the parameters in the dataset with complicated relationships among illnesses, hospitalizations, and deaths caused by foodborne outbreaks, the regression models provide biased results. The consideration of causality demonstrates that some serotype categories have a strong effect on illnesses, and some result in hospitalization and/or death.

## 5. Conclusions

Foodborne diseases caused by *Salmonella* spp. are some of the leading public concerns worldwide despite all efforts to reduce the number of outbreaks [27]. The negative binomial regression model performed for analysis revealed that single serotypes and certain IFSAC food categories showed significant effects on the numbers of illnesses, hospitalizations, and deaths caused by foodborne outbreaks of *Salmonella* in the present study. Also, the results indicated statistically validated causal relationships between *Salmonella* serotypes and death counts with the mediation of illness and hospitalization counts in related foodborne outbreaks. Understanding the relationships between *Salmonella* serotypes and specific food commodities is important to prevent foodborne diseases since the frequencies of certain serotypes as Enteritidis, Typhimurium, and Newport are higher compared with other known outbreak serotypes. *Salmonella* has historically been a concern in animal food products, and this trend has persisted as evidenced by our study. However, fruits and vegetables have also been involved in *Salmonella* outbreaks more commonly in recent years, indicating produce safety at the farm level should be one of the main concerns to address by public health officials and researchers. Public health officials may benefit from descriptive and analytical results of this study and reach conclusions faster during the investigation of foodborne outbreaks linked to *Salmonella* serotypes. For future studies, foodborne outbreak data provided by NORS or similar surveillance systems can be analyzed with more inputs as the location, time of the year, and environmental factors (temperature and precipitation) with artificial intelligence and machine learning tools. Prediction and risk assessment applications can be developed for officials working in the field during the investigation of foodborne outbreaks.

## Figures and Tables

**Table 1:** Ratio scales of causal mediation analysis (causal effect) with hospitalization and illness counts related to foodborne outbreaks caused by *Salmonella* serotypes as mediators.

Scale	Parameter *	Formula
Rate	$R^{CDE}$	$E\left[Y_{am}\right]/E\left[Y_{a^{*}m}\right]$
	$R^{PNDE}$	$E\left[Y_{aM_{a^{*}}}\right]/E\left[Y_{a^{*}M_{a^{*}}}\right]$
	$R^{TNDE}$	$E\left[Y_{aM_{a}}\right]/E\left[Y_{a^{*}M_{a}}\right]$
	$R^{PNIE}$	$E\left[Y_{a^{*}M_{a}}\right]/E\left[Y_{a^{*}M_{a^{*}}}\right]$
	$R^{TNIE}$	$E\left[Y_{aM_{a}}\right]/E\left[Y_{aM_{a^{*}}}\right]$
	$R^{TE}$	$R^{PNDE}\times R^{TNIE}$ $\;\mathrm{or}\;R^{TNDE}\times R^{PNIE}$
Excess relative rate	${ER}^{CDE}$	$E\left[Y_{am}-Y_{a^{*}m}\right]/E\left[Y_{a^{*}M_{a^{*}}}\right]$
	${ER}^{{INT}_{ref}}$	$R^{PNDE}-1-{ER}^{CDE}$
	${ER}^{{INT}_{med}}$	$R^{TNIE}*R^{PNDE}-R^{PNDE}-R^{PNIE}+1$
	${ER}^{PNIE}$	$R^{PNIE}-1$
Proportion excess relative rate	${prop}^{{ER}^{CDE}}$	${ER}^{CDE}/\left(R^{TE}-1\right)$
	${prop}^{{ER}^{{INT}_{ref}}}$	${ER}^{{INT}_{ref}}/\left(R^{TE}-1\right)$
	${prop}^{{ER}^{{INT}_{med}}}$	${ER}^{{INT}_{med}}/\left(R^{TE}-1\right)$
	${prop}^{{ER}^{PNIE}}$	${ER}^{PNIE}/\left(R^{TE}-1\right)$
Overall	PM	$\left(R^{PNDE}*\left(R^{TNIE}-1\right)\right)/\left(R^{TE}-1\right)$
	INT	$\left({ER}^{{INT}_{ref}}+{ER}^{{INT}_{med}}\right)/\left(R^{TE}-1\right)$
	PE	$\left({ER}^{{INT}_{ref}}+{ER}^{{INT}_{med}}+{ER}^{PNIE}\right)/\left(R^{TE}-1\right)$

* *R^CDE^*, controlled direct effect rate ratio; *R^PNDE^*, pure natural direct effect rate ratio; *R^TNDE^*, total natural direct effect rate ratio; *R^PNIE^*, pure natural indirect effect rate ratio; *R^TNIE^*, total natural indirect effect rate ratio; *R^TE^*, total effect rate ratio; *ER^CDE^*, excess relative rate due to controlled direct effect; *ER^INTref^*, excess relative rate due to reference interaction; *ER^INTmed^*, excess relative rate due to mediated interaction; *ER^PNIE^*, excess relative rate due to pure natural indirect effect; *ER^CDE^(prop)*, proportion *ER^CDE^*; *ER^INTref^(prop)*, proportion *ER^INTref^*; *ER^INTmed^(prop)*, proportion *ER^INTme^*; *ER^PNIE^(prop)*, proportion *ER^PNIE^; PM*, overall proportion mediated; *INT*, overall proportion attributable to interaction; *PE*, overall proportion eliminated.

**Table 2:** Frequency of foodborne outbreaks in the United States associated with *Salmonella* spp. between 1998 and 2021 listed by the United States Center for Disease Control and Prevention National Outbreak Reporting System (NORS) database with confirmed pathogen.

Serotype	Number of Outbreaks (%)	Illnesses (%)	Hospitalization (%)	Death (%)
Enteritidis	815 (28.42)	20,599 (25.12)	1981 (17.98)	18 (15.65)
Typhimuirum	359 (12.52)	9202 (11.22)	1232 (11.18)	20 (17.39)
Newport	220 (7.67)	8036 (9.80)	1258 (11.42)	14 (12.17)
* Other	203 (7.08)	5036 (6.14)	739 (6.71)	10 (8.70)
Heidelberg.	165 (5.75)	5653 (6.89)	863 (7.83)	10 (8.70)
Unknown	139 (4.85)	1943 (2.37)	176 (1.60)	2 (1.74)
Javiana	95 (3.31)	3923 (4.78)	533 (4.84)	5 (4.35)
I 4,[5],12:i:-	88 (3.07)	2078 (2.53)	405 (3.68)	5 (4.35)
Braenderup	73 (2.55)	1377 (1.68)	226 (2.05)	1 (0.87)
Multiple	73 (2.55)	5288 (6.45)	678 (6.15)	6 (5.22)
Infantis	67 (2.34)	1697 (2.07)	230 (2.09)	1 (0.87)
Montevideo	61 (2.13)	1913 (2.33)	224 (2.03)	1 (0.87)
Thompson	56 (1.95)	1256 (1.53)	114 (1.03)	1 (0.87)
Saintpaul	53 (1.85)	3086 (3.76)	616 (5.59)	2 (1.74)
Muenchen	42 (1.46)	1164 (1.42)	96 (0.87)	2 (1.74)
Oranienburg	39 (1.36)	1733 (2.11)	388 (3.52)	1 (0.87)
Hadar	25 (0.87)	565 (0.69)	87 (0.79)	0
Berta	25 (0.87)	583 (0.71)	59 (0.54)	1 (0.87)
Group B	23 (0.80)	413 (0.50)	48 (0.44)	0
Paratyphi B	22 (0.77)	447 (0.55)	41 (0.37)	0
Agona	22 (0.77)	598 (0.73)	102 (0.93)	0
Anatum	18 (0.63)	465 (0.57)	41 (0.37)	1 (0.87)
Uganda	18 (0.63)	362 (0.44)	64 (0.58)	0
Hartford	15 (0.52)	250 (0.30)	23 (0.21)	0
Schwarzengrund	15 (0.52)	223 (0.27)	37 (0.34)	1 (0.87)
Stanley	15 (0.52)	237 (0.29)	28 (0.25)	0
Weltevreden	15 (0.52)	184 (0.22)	22 (0.20)	0
Miami	14 (0.49)	342 (0.42)	76 (0.69)	1 (0.87)
Bareilly	13 (0.45)	183 (0.22)	20 (0.18)	0
Brandenburg	13 (0.45)	137 (0.17)	30 (0.27)	0
Mbandaka	13 (0.45)	549 (0.67)	59 (0.54)	0
Reading	13 (0.45)	537 (0.65)	142 (1.29)	1 (0.87)
Wirchow	11 (0.38)	220 (0.27)	21 (0.19)	0
Baildon	10 (0.35)	505 (0.62)	52 (0.47)	3 (2.61)
Poona	10 (0.35)	1097 (1.34)	244 (2.21)	8 (6.96)
Typhi	10 (0.35)	115 (0.14)	63 (0.57)	0
All	2868 (100.00)	81,996 (100.00)	11,018 (100.00)	115 (100.00)

* The total number of outbreaks related to serotypes causing fewer than 10 outbreaks was pooled as “other”.

**Table 3:** Frequency of foodborne outbreaks in the United States associated with *Salmonella* spp. associated with the Interagency Food Safety Analytics Collaboration (IFSAC) food categories between 1998 and 2021 listed by the United States Center for Disease Control and Prevention National Outbreak Reporting System (NORS) database with confirmed pathogens.

IFSAC Category	Number of Outbreaks (%)	Illnesses (%)	Hospitalization (%)	Death (%)
Uncategorized	1308 (45.61)	22,338 (27.24)	3000 (27.23)	38 (33.04)
Multiple	617 (21.51)	18,613 (22.70)	2029 (18.42)	13 (11.30)
Chicken	177 (6.17)	5250 (6.40)	809 (7.34)	6 (5.22)
Eggs	160 (5.58)	5870 (7.16)	400 (3.63)	3 (2.61)
Pork	99 (3.45)	3004 (3.66)	401 (3.64)	4 (3.48)
Fruits	83 (2.89)	4279 (5.22)	798 (7.24)	18 (15.65)
Beef	81 (2.82)	2703 (3.30)	448 4.07)	3 (2.61)
Turkey	65 (2.27)	2685 (3.27)	360 (3.27)	3 (2.61)
Seeded vegetables	59 (2.06)	6099 (7.44)	1113 (10.10)	13 (11.30)
Dairy	43 (1.50)	1206 (1.47)	204 (1.85)	1 (0.87)
Sprouts	39 (1.36)	1667 (2.03)	159 (1.44)	2 (1.74)
* Other	29 (1.01)	846 (1.03)	156 (1.42)	0
Nuts-seeds	22 (0.77)	1954 (2.38)	347 (3.15)	11 (9.57)
Vegetable row crops	22 (0.77)	962 (1.17)	69 (0.63)	0
Fish	21 (0.73)	959 (1.17)	91 (0.83)	0
Crustaceans	11 (0.38)	149 (0.18)	30 (0.27)	0
Herbs	11 (0.38)	571 (0.70)	83 (0.75)	0
Other meat	11 (0.38)	196 (0.24)	21 (0.19)	0
Root/Underground	10 (0.35)	2645 (3.23)	500 (4.54)	0
All	2868 (100.00)	81,996 (100.00)	11,018 (100.00)	115 (100.00)

* The total number of outbreaks related to IFSAC food categories causing fewer than 10 outbreaks was pooled as “other”.

**Table 4:** The frequencies (percentages) of *Salmonella* serotypes for illness, hospitalization, and death counts related to foodborne outbreaks (n = 2868).

	Frequency of Outbreaks (%)				
Variable	[0, 5)	[5, 10)	[10, 100)	[100, 1000)	(≥1000)
Illness	620 (21.62%)	719 (25.07%)	1386 (48.33%)	139 (4.85%)	4 (0.14%)
Hospitalization	2340 (81.59%)	308 (10.74%)	209 (7.29%)	11 (0.38%)	
Death	2866 (99.93%)	2 (0.07%)			

**Table 5:** The magnitudes of the calculated dispersion parameters, test statistics, and corresponding *p*-values for death, illness, and hospitalization counts related to foodborne outbreaks caused by *Salmonella* serotypes.

Variables	Dispersion	Test Statistics	*p*-Value
Illness	134.5764	3.4620	0.000 ***
Hospitalization	15.7473	5.8027	0.000 ***
Death	1.2177	2.3156	0.010 *

Significance codes: 0, ***; 0.001, *; 0.05 ‘.’.

**Table 6:** Negative binomial regression model summary for death, illness, and hospitalization counts caused by outbreaks of *Salmonella* serotypes and associated Interagency Food Safety Analytics Collaboration (IFSAC) food categories between 1998 and 2021 listed by the United States Center for Disease Control and Prevention National Outbreak Reporting System (NORS) database with confirmed pathogens.

	Variable (*p*-Value)		
	Illness	Hospitalization	Death
Serotype	Baildon (0.061.)	Heidelberg (0.000 ***)	Baildon (0.026 *)
	Braenderup (0.018 *)	I 4,[5],12:i:- (0.001 **)	Heidelberg (0.063.)
	Heidelberg (0.000 ***)	Infantis (0.056.)	Saintpaul (0.000 ***)
	Javiana (0.000 ***)	Javiana (0.001 **)	
	Montevideo (0.029 *)	Miami (0.077.)	
	Multiple (0.000 ***)	Multiple (0.000 ***)	
	Saintpaul (0.010 *)	Newport (0.000 ***)	
	Unknown (0.000 ***)	Oranienburg (0.022 *)	
	Weltevreden (0.091.)	Poona (0.012 *)	
		Reading (0.003 **)	
		Saintpaul (0.000 ***)	
		Typhimurium (0.028 *)	
		Unknown (0.000 ***)	
IFSAC food category	Beef (0.000 ***)	Beef (0.000 ***)	Fruits (0.001 **)
	Chicken (0.000 ***)	Chicken (0.000 ***)	Multiple (0.048 *)
	Dairy (0.007 **)	Dairy (0.011 *)	
	Eggs (0.000 ***)	Eggs (0.076.)	
	Fish (0.002 **)	Fruits (0.000 ***)	
	Fruits (0.000 ***)	Herbs (0.007 **)	
	Herbs (0.002 **)	Multiple (0.000 ***)	
	Multiple (0.000 ***)	Nuts-seeds (0.000 ***)	
	Nuts-seeds (0.000 ***)	Other (0.015 *)	
	Other (0.022 *)	Pork (0.002 **)	
	Pork (0.000 ***)	Root/underground (0.000 ***)	
	Root/underground (0.000 ***)	Seeded vegetables (0.000 ***)	
	Seeded vegetables (0.000 ***)	Sprouts (0.035)	
	Sprouts (0.000 ***)	Turkey (0.000 ***)	
	Turkey (0.000 ***)		
	Vegetable row crops (0.000 ***)		

Significance codes: 0, ***; 0.001, **; 0.01, *; 0.05 ‘.’.

## Data Availability

The data that support the findings of this study are available from the corresponding author upon reasonable request.

## Supplementary Materials

The following supporting information can be downloaded at: https://www.mdpi.com/article/10.3390/pathogens13121134/s1, Table S1: Negative Binomial regression model summary for illness counts related to foodborne outbreaks caused by *Salmonella* serotypes (Mediator 1); Table S2: Negative Binomial regression model summary for hospitalization counts related to foodborne outbreaks caused by *Salmonella* serotypes (Mediator 2); Table S3: Negative Binomial regression model summary for death counts related to foodborne outbreaks caused by *Salmonella* serotypes; Table S4: Result of causal mediation analysis with hospitalization and illness counts related to foodborne outbreaks caused by *Salmonella* serotypes as mediators.

## References

[1] Scallan E., Hoekstra R.M., Angulo F.J., Tauxe R.V., Widdowson M.-A., Roy S.L., Jones J.L., Griffin P.M. (2011). Foodborne Illness Acquired in the United States—Major Pathogens. Emerg. Infect. Dis..

[2] Jackson B.R., Griffin P.M., Cole D., Walsh K.A., Chai S.J. (2013). Outbreak-Associated *Salmonella enterica* Serotypes and Food Commodities, United States, 1998–2008. Emerg. Infect. Dis..

[3] Canning M., Birhane M.G., Dewey-Mattia D., Lawinger H., Cote A., Gieraltowski L., Schwensohn C., Tagg K.A., Francois Watkins L.K., Park Robyn M. (2023). *Salmonella* Outbreaks Linked to Beef, United States, 2012–2019. J. Food Prot..

[4] Crowe S.J., Mahon B.E., Vieira A.R., Gould L.H. (2015). Vital Signs: Multistate Foodborne Outbreaks—United States, 2010–2014. MMWR Morb. Mortal. Wkly. Rep..

[5] Gould L.H., Walsh K.A., Vieira A.R., Herman K., Williams I.T., Hall A.J., Cole D., Centers for Disease Control and Prevention (2013). Surveillance for Foodborne Disease Outbreaks-United States, 1998–2008. Morb. Mortal. Wkly. Report. Surveill. Summ..

[6] Marus J.R., Magee M.J., Manikonda K., Nichols M.C. (2019). Outbreaks of *Salmonella enterica* Infections Linked to Animal Contact: Demographic and Outbreak Characteristics and Comparison to Foodborne Outbreaks—United States, 2009–2014. Zoonoses Public Health.

[7] Snyder T.R., Boktor S.W., M’ikanatha N.M. (2019). Salmonellosis Outbreaks by Food Vehicle, Serotype, Season, and Geographical Location, United States, 1998 to 2015. J. Food Prot..

[8] Cheng R.A., Eade C.R., Wiedmann M. (2019). Embracing Diversity: Differences in Virulence Mechanisms, Disease Severity, and Host Adaptations Contribute to the Success of Nontyphoidal Salmonella as a Foodborne Pathogen. Front. Microbiol..

[9] Dewey-Mattia D., Manikonda K., Hall A.J., Wise M.E., Crowe S.J. (2018). Surveillance for Foodborne Disease Outbreaks—United States, 2009–2015. MMWR Surveill. Summ..

[10] Jones T.F., Ingram L.A., Cieslak P.R., Vugia D.J., Tobin-D’Angelo M., Hurd S., Medus C., Cronquist A., Angulo F.J. (2008). Salmonellosis Outcomes Differ Substantially by Serotype. J. Infect. Dis..

[11] O’Rourke H.P., Vazquez E. (2019). Mediation Analysis with Zero-Inflated Substance Use Outcomes: Challenges and Recommendations. Addict. Behav..

[12] Cheng J., Cheng N.F., Guo Z., Gregorich S., Ismail A.I., Gansky S.A. (2017). Mediation Analysis for Count and Zero-Inflated Count Data. Stat. Methods Med. Res..

[13] Thomson J.L., Landry A.S., Walls T.I. (2024). Direct and Indirect Effects of Food and Nutrition Security on Dietary Choice and Healthfulness of Food Choice: Causal Mediation Analysis. Curr. Dev. Nutr..

[14] Xu S., Coffman D.L., Luta G., Niaura R.S. (2023). Tutorial on Causal Mediation Analysis with Binary Variables: An Application to Health Psychology Research. Health Psychol..

[15] Zhang Z., Zheng C., Kim C., Van Poucke S., Lin S., Lan P. (2016). Causal Mediation Analysis in the Context of Clinical Research. Ann. Transl. Med..

[16] Hall A.J., Wikswo M.E., Manikonda K., Roberts V.A., Yoder J.S., Gould L.H. (2013). Acute Gastroenteritis Surveillance through the National Outbreak Reporting System, United States. Emerg. Infect. Dis..

[17] Dewey-Mattia D., Roberts V.A., Vieira A., Fullerton K.E. (2016). Foodborne (1973–2013) and Waterborne (1971–2013) Disease Outbreaks—United States. MMWR Morb. Mortal. Wkly. Rep..

[18] Nsoesie E.O., Kluberg S.A., Brownstein J.S. (2014). Online Reports of Foodborne Illness Capture Foods Implicated in Official Foodborne Outbreak Reports. Prev. Med..

[19] White A.E., Tillman A.R., Hedberg C., Bruce B.B., Batz M., Seys S.A., Dewey-Mattia D., Bazaco M.C., Walter E.S. (2022). Foodborne Illness Outbreaks Reported to National Surveillance, United States, 2009–2018. Emerg. Infect. Dis..

[20] Valeri L., Vanderweele T.J. (2013). Mediation Analysis Allowing for Exposure-Mediator Interactions and Causal Interpretation: Theoretical Assumptions and Implementation with SAS and SPSS Macros. Psychol. Methods.

[21] VanderWeele T., Vansteelandt S. (2014). Mediation Analysis with Multiple Mediators. Epidemiol. Methods.

[22] VanderWeele T.J. (2015). Explanation in Causal Inference: Methods for Mediation and Interaction.

[23] Chin H.C., Quddus M.A. (2003). Modeling Count Data with Excess Zeroes. Sociol. Methods Res..

[24] Venables W.N., Ripley B.D. (2002). Modern Applied Statistics with S.

[25] BS1125 (2021). GitHub-BS1125/CMAverse: A Suite of Functions for Reproducible Causal Mediation Analyses.

[26] R Core Team R: A Language and Environment for Statistical Computing. R Foundation for Statistical Computing. https://www.r-project.org/.

[27] Popa G.L., Papa M.I. (2021). *Salmonella* Spp. Infection–A Continuous Threat Worldwide. GERMS.

[28] Xiang Y., Li F., Dong N., Tian S., Zhang H., Du X., Zhou X., Xu X., Yang H., Xie J. (2020). Investigation of a Salmonellosis Outbreak Caused by Multidrug Resistant *Salmonella* Typhimurium in China. Front. Microbiol..

[29] Alt K., Simon S., Helmeke C., Kohlstock C., Prager R., Tietze E., Rabsch W., Karagiannis I., Werber D., Frank C. (2015). Outbreak of Uncommon O4 Non-Agglutinating *Salmonella* Typhimurium Linked to Minced Pork, Saxony-Anhalt, Germany, January to April 2013. PLoS ONE.

[30] Folster J.P., Grass J.E., Bicknese A., Taylor J., Friedman C.R., Whichard J.M. (2017). Characterization of Resistance Genes and Plasmids from Outbreaks and Illness Clusters Caused by *Salmonella* Resistant to Ceftriaxone in the United States, 2011–2012. Microb. Drug Resist..

[31] Nichols M., Gollarza L., Sockett D., Aulik N., Patton E., Francois Watkins L.K., Gambino-Shirley K.J., Folster J.P., Chen J.C., Tagg K.A. (2022). Outbreak of Multidrug-Resistant *Salmonella* Heidelberg Infections Linked to Dairy Calf Exposure, United States, 2015–2018. Foodborne Pathog. Dis..

[32] Punchihewage-Don A.J., Hawkins J., Adnan A.M., Hashem F., Parveen S. (2022). The Outbreaks and Prevalence of Antimicrobial Resistant *Salmonella* in Poultry in the United States: An Overview. Heliyon.

[33] Brown A.C., Grass J.E., Richardson L.C., Nisler A.L., Bicknese A.S., Gould L.H. (2016). Antimicrobial Resistance in *Salmonella* That Caused Foodborne Disease Outbreaks: United States, 2003–2012. Epidemiol. Infect..

[34] Barton Behravesh C., Mody R.K., Jungk J., Gaul L., Redd J.T., Chen S., Cosgrove S., Hedican E., Sweat D., Chávez-Hauser L. (2011). 2008 Outbreak of *Salmonella* Saintpaul Infections Associated with Raw Produce. N. Engl. J. Med..

[35] Taylor E., Kastner J., Renter D. (2010). Challenges Involved in the *Salmonella* Saintpaul Outbreak and Lessons Learned. J. Public Health Manag. Pract..

[36] Chen R., Cheng R.A., Wiedmann M., Orsi R.H. (2022). Development of a Genomics-Based Approach to Identify Putative Hypervirulent Nontyphoidal *Salmonella* Isolates: *Salmonella enterica* Serovar Saintpaul as a Model. mSphere.

[37] McGeoch L.J., Hoban A., Sawyer C., Rabie H., Painset A., Browning L., Brown D., McCarthy C., Nelson A., Firme A. (2024). *Salmonella* Saintpaul Outbreak Associated with Cantaloupe Consumption, the United Kingdom and Portugal, September to November 2023. Epidemiol. Infect..

[38] Munnoch S.A., Ward K., Sheridan S., Fitzsimmons G.J., Shadbolt C.T., Piispanen J.P., Wang Q., Ward T.J., Worgan T.L.M., Oxenford C. (2009). A Multi-State Outbreak of *Salmonella* Saintpaul in Australia Associated with Cantaloupe Consumption. Epidemiol. Infect..

[39] Laughlin M., Bottichio L., Weiss J., Higa J., McDonald E., Sowadsky R., Fejes D., Saupe A., Provo G., Seelman S. (2019). Multistate Outbreak of *Salmonella* Poona Infections Associated with Imported Cucumbers, 2015–2016. Epidemiol. Infect..

[40] European Centre for Disease Prevention and Control, European Food Safety Authority (2019). Multi-Country Outbreak of *Salmonella* Poona Infections Linked to Consumption of Infant Formula. EFSA Support. Publ..

[41] Morasi R.M., Rall V.L.M., Dantas S.T.A., Alonso V.P.P., Silva N.C.C. (2022). *Salmonella* spp. In Low Water Activity Food: Occurrence, Survival Mechanisms, and Thermoresistance. J. Food Sci..

[42] Basler C., Nguyen T.-A., Anderson T.C., Hancock T., Behravesh C.B. (2016). Outbreaks of Human *Salmonella* Infections Associated with Live Poultry, United States, 1990–2014. Emerg. Infect. Dis..

[43] Moffatt C.R., Musto J. (2013). *Salmonella* and Egg-Related Outbreaks. Microbiol. Aust..

[44] Cardoso M.J., Nicolau A.I., Borda D., Nielsen L., Maia R.L., Møretrø T., Ferreira V., Knøchel S., Langsrud S., Teixeira P. (2021). *Salmonella* in Eggs: From Shopping to Consumption—A Review Providing an Evidence-Based Analysis of Risk Factors. Compr. Rev. Food Sci. Food Saf..

[45] Chousalkar K., Gast R., Martelli F., Pande V. (2017). Review of Egg-Related Salmonellosis and Reduction Strategies in United States, Australia, United Kingdom and New Zealand. Crit. Rev. Microbiol..

[46] Hanning I.B., Nutt J.D., Ricke S.C. (2009). Salmonellosis Outbreaks in the United States due to Fresh Produce: Sources and Potential Intervention Measures. Foodborne Pathog. Dis..

[47] Topalcengiz Z., Danyluk M.D. (2019). Fate of Generic and Shiga Toxin-Producing Escherichia Coli (STEC) in Central Florida Surface Waters and Evaluation of EPA Worst Case Water as Standard Medium. Food Res. Int..

[48] Topalcengiz Z., Mcegan R., Danyluk M.D. (2019). Fate of *Salmonella* in Central Florida Surface Waters and Evaluation of EPA Worst Case Water as a Standard Medium. J. Food Prot..

[49] Topalcengiz Z., Jeamsripong S., Spanninger P., Persad A., Wang F., Buchanan R.L., LeJeune J., Kniel K.E., Jay-Russell M., Danyluk M.D. (2020). Survival of Shiga Toxin–Producing *Escherichia coli* in Various Wild Animal Feces That May Contaminate Produce. J. Food Prot..

[50] Topalcengiz Z., Spanninger P., Jeamsripong S., Persad A., Buchanan R.L., Saha J., LeJeune J., Jay-Russell M., Kniel K.E., Danyluk M.D. (2020). Survival of *Salmonella* in Various Wild Animal Feces That May Contaminate Produce. J. Food Prot..

[51] Dyda A., Nguyen P.Y., Chughtai A.A., MacIntyre C.R. (2020). Changing Epidemiology of *Salmonella* Outbreaks Associated with Cucumbers and Other Fruits and Vegetables. Glob. Biosecurity.

[52] Callejón R.M., Rodríguez-Naranjo M.I., Ubeda C., Hornedo-Ortega R., Garcia-Parrilla M.C., Troncoso A.M. (2015). Reported Foodborne Outbreaks due to Fresh Produce in the United States and European Union: Trends and Causes. Foodborne Pathog. Dis..

[53] Krishnasamy V.P., Marshall K., Dewey-Mattia D., Wise M. (2019). Outbreak Characteristics and Epidemic Curves for Multistate Outbreaks of *Salmonella* Infections Associated with Produce: United States, 2009–2015. Foodborne Pathog. Dis..

[54] Laufer A.S., Grass J., Holt K., Whichard J.M., Griffin P.M., Gould L.H. (2014). Outbreaks of *Salmonella* Infections Attributed to Beef–United States, 1973–2011. Epidemiol. Infect..

[55] Strickland A.J., Sampedro F., Hedberg C.W. (2023). Quantitative Risk Assessment of *Salmonella* in Ground Beef Products and the Resulting Impact of Risk Mitigation Strategies on Public Health. J. Food Prot..

[56] Hilbe J.M. (2011). Negative Binomial Regression.

[57] Benjamin L., Atwill E.R., Jay-Russell M., Cooley M., Carychao D., Gorski L., Mandrell R.E. (2013). Occurrence of Generic *Escherichia coli, E. coli* O157 and *Salmonella* Spp. In Water and Sediment from Leafy Green Produce Farms and Streams on the Central California Coast. Int. J. Food Microbiol..

[58] Buyrukoğlu G., Buyrukoğlu S., Topalcengiz Z. (2021). Comparing Regression Models with Count Da-ta to Artificial Neural Network and Ensemble Models for Prediction of Generic *Escherichia coli* Population in Agricultural Ponds Based on Weather Station Measurements. Microb. Risk Anal..

