# Supplementary material for: Causal Mediation Analysis of Foodborne Salmonella Outbreaks in the United States: Serotypes and Food Vehicles
Source: Pathogens. 2024 Dec 22;13(12):1134. doi: 10.3390/pathogens13121134 (PMC11676911; doi:10.3390/pathogens13121134)
Supplement: Supplementary file 1 [file pathogens-13-01134-s001.zip › pathogens-3353873-supplementary.pdf]

Supplements

Table S1. Negative Binomial regression model summary for illness counts related to foodborne outbreaks caused by *Salmonella* serotypes (Mediator 1).

	Variables	Estimates	Standard Error	Z-Score	*P*-value
	(Intercept)	2.764	0.048	57.493	0.000***
Serotype	Agona	0.109	0.242	0.449	0.653
	Anatum	-0.067	0.266	-0.252	0.801
	Baildon	0.658	0.352	1.867	0.061.
	Bareilly	-0.237	0.315	-0.751	0.453
	Berta	0.055	0.228	0.240	0.810
	Braenderup	-0.326	0.138	-2.363	0.018*
	Brandenburg	-0.488	0.318	-1.536	0.124
	Group B	-0.103	0.236	-0.436	0.662
	Hadar	-0.130	0.229	-0.568	0.570
	Hartford	-0.315	0.293	-1.075	0.283
	Heidelberg	0.324	0.095	3.404	0.000***
	I 4,[5],12:i:-	-0.058	0.127	-0.459	0.646
	Infantis	0.175	0.143	1.228	0.219
	Javiana	0.477	0.123	3.881	0.000***
	Mbandaka	0.453	0.313	1.447	0.148
	Miami	0.221	0.301	0.736	0.462
	Montevideo	0.323	0.149	2.172	0.029*
	Muenchen	-0.025	0.177	-0.139	0.889
	Multiple	0.948	0.138	6.914	0.000***
	Newport	0.121	0.088	1.375	0.169
	Oranienburg	-0.022	0.184	-0.119	0.905
	Other	-0.010	0.090	-0.112	0.911
	Paratyphi B	-0.299	0.246	-1.217	0.223
	Poona	0.554	0.355	1.560	0.118
	Reading	0.394	0.313	1.258	0.209
	Saintpaul	0.412	0.160	2.568	0.010*
	Schwarzengrund	-0.280	0.293	-0.958	0.338
	Stanley	-0.328	0.294	-1.113	0.266
	Thompson	-0.100	0.155	-0.648	0.517
	Typhi	-0.479	0.360	-1.329	0.184
	Typhimurium	0.098	0.073	1.343	0.179
	Uganda	-0.289	0.271	-1.065	0.287
	Unknown	-0.366	0.104	-3.537	0.000***
	Virchow	0.007	0.340	0.022	0.983
	Weltevreden	-0.509	0.301	-1.692	0.091.
IFSAC category	Beef	0.661	0.128	5.177	0.000***
	Chicken	0.519	0.089	5.778	0.000***

	Crustaceans	-0.190	0.347	-0.549	0.583
	Dairy	0.468	0.174	2.686	0.007**
	Eggs	0.817	0.097	8.408	0.000***
	Fish	0.764	0.248	3.082	0.002**
	Fruits	0.936	0.128	7.340	0.000***
	Herbs	0.932	0.336	2.787	0.002**
	Multiple	0.553	0.055	10.049	0.000***
	Nuts-Seeds	1.649	0.239	6.883	0.000***
	Other	0.473	0.211	2.245	0.025*
	Other Meat	0.069	0.341	0.202	0.840
	Pork	0.563	0.119	4.723	0.000***
	Root/Underground	2.714	0.351	7.727	0.000***
	Seeded Vegetables	1.589	0.151	10.557	0.000***
	Sprouts	0.825	0.182	4.529	0.000***
	Turkey	0.827	0.144	5.735	0.000***
	Vegetable Row Crops	0.946	0.278	3.970	0.000***
	Null deviance:	3982.8	2867 degrees of freedom		
	Residual deviance:	3254.4	2814 degrees of freedom		
	AIC:	24260			
	2 x log-likelihood:	-24150.442			

Significance codes: 0 ‘***’ 0.001 ‘**’ 0.01 ‘*’ 0.05 ‘.’ 0.1 ‘ ’ 1.

Table S2. Negative Binomial regression model summary for hospitalization counts related to foodborne outbreaks caused by *Salmonella* serotypes (Mediator 2).

	Variables	Estimates	Standard Error	Z-Score	*P*-value
	(Intercept)	0.618	0.066	9.439	0.000***
Serotype	Agona	0.590	0.315	1.872	0.061.
	Anatum	-0.277	0.364	-0.761	0.447
	Baildon	0.429	0.462	0.929	0.353
	Bareilly	-0.219	0.438	-0.499	0.618
	Berta	-0.131	0.314	-0.417	0.677
	Braenderup	0.082	0.184	0.444	0.657
	Brandenburg	0.181	0.423	0.429	0.668
	Group B	0.010	0.319	0.030	0.976
	Hadar	0.177	0.304	0.582	0.561
	Hartford	-0.513	0.419	-1.226	0.220
	Heidelberg	0.686	0.125	5.502	0.000***
	I 4,[5],12:i:-	0.546	0.166	3.290	0.001**
	Infantis	0.361	0.189	1.908	0.056.
	Javiana	0.531	0.161	3.290	0.001**
	Mbandaka	0.358	0.412	0.868	0.385
	Miami	0.691	0.391	1.769	0.077.
	Montevideo	0.292	0.198	1.473	0.141
	Muenchen	-0.137	0.243	-0.565	0.572
	Multiple	0.815	0.178	4.584	0.000***
	Newport	0.434	0.117	3.725	0.000***
	Oranienburg	0.548	0.240	2.288	0.022*
	Other	0.165	0.120	1.371	0.170
	Paratyphi B	-0.519	0.344	-1.508	0.132
	Poona	1.129	0.451	2.500	0.012*
	Reading	1.183	0.399	2.967	0.003**
	Saintpaul	0.973	0.206	4.730	0.000***
	Schwarzengrund	0.015	0.394	0.0438	0.969
	Stanley	-0.315	0.411	-0.765	0.445
	Thompson	-0.214	0.213	-1.003	0.316
	Typhi	0.997	0.454	2.196	0.028*
	Typhimurium	0.220	0.098	2.246	0.025*
	Uganda	0.021	0.361	0.057	0.954
	Unknown	-0.627	0.149	-4.222	0.000***
	Virchow	-0.426	0.474	-0.899	0.368
	Weltevreden	-0.452	0.424	-1.065	0.287
IFSAC category	Beef	0.796	0.166	4.794	0.000***
	Chicken	0.598	0.119	5.046	0.000***

	Crustaceans	0.284	0.464	0.613	0.540
	Dairy	0.577	0.228	2.529	0.011*
	Eggs	0.231	0.132	1.777	0.076.
	Fish	0.512	0.329	1.555	0.120
	Fruits	1.222	0.163	7.479	0.000***
	Herbs	1.171	0.431	2.717	0.007**
	Multiple	0.362	0.074	4.899	0.000***
	Nuts-Seeds	1.949	0.304	6.422	0.000***
	Other	0.672	0.275	2.446	0.015*
	Other Meat	-0.211	0.473	-0.447	0.655
	Pork	0.495	0.157	3.157	0.002**
	Root/Underground	2.793	0.440	6.347	0.000***
	Seeded Vegetables	1.638	0.191	8.565	0.000***
	Sprouts	0.505	0.240	2.110	0.035
	Turkey	0.649	0.189	3.431	0.000***
	Vegetable Row Crops	0.413	0.318	1.301	0.193
	Null deviance:	3627.7	2867 degrees of freedom		
	Residual deviance:	2963.9	2814 degrees of freedom		
	AIC:	12834			
	2 x log-likelihood:	-12723.837			

Significance codes: 0 ‘***’ 0.001 ‘**’ 0.01 ‘*’ 0.05 ‘.’ 0.1 ‘ ’ 1.

Table S3. Negative Binomial regression model summary for death counts related to foodborne outbreaks caused by *Salmonella* serotypes.

	Variables	Estimates	Standard Error	Z-Score	*P*-value
	(Intercept)	-3.821	0.295	-12.959	0.000***
Serotype	Agona	-23.197	79363.368	0.000	0.999
	Anatum	0.456	1.347	0.339	0.735
	Baildon	2.156	0.965	2.234	0.026*
	Bareilly	-23.816	108290.065	0.000	0.999
	Berta	0.385	1.123	0.343	0.732
	Braenderup	-0.661	1.067	-0.619	0.536
	Brandenburg	-23.067	110005.084	0.000	0.999
	Group B	-22.872	81624.931	0.000	0.999
	Hadar	-22.853	78953.628	0.000	0.999
	Hartford	-22.868	102307.032	0.000	0.999
	Heidelberg	0.857	0.460	1.862	0.063.
	I 4,[5],12:i:-	0.039	0.460	0.053	0.958
	Infantis	-0.602	1.119	-0.538	0.591
	Javiana	0.369	0.632	0.584	0.559
	Mbandaka	-23.108	105811.727	0.000	0.999
	Miami	0.637	1.293	0.493	0.622
	Montevideo	-0.297	1.077	-0.276	0.773
	Muenchen	0.547	0.819	0.668	0.504
	Multiple	-0.464	0.777	-0.598	0.550
	Newport	0.620	0.446	1.390	0.164
	Oranienburg	-0.001	1.088	-0.001	0.999
	Other	0.446	0.465	0.959	0.338
	Paratyphi B	-22.675	78059.682	0.000	0.999
	Poona	0.706	1.131	0.624	0.533
	Reading	-1.506	2.320	-0.649	0.516
	Saintpaul	-9.972	2.710	-3.679	0.000***
	Schwarzengrund	0.929	1.248	0.744	0.457
	Stanley	-22.893	97638.801	0.000	0.999
	Thompson	-0.463	1.170	-0.395	0.693
	Typhi	-23.245	116835.132	0.000	0.999
	Typhimurium	0.065	0.442	0.147	0.883
	Uganda	-23.507	85800.147	0.000	0.999
	Unknown	-0.321	0.774	-0.415	0.678
	Virchow	-22.883	109953.152	0.000	0.999
	Weltevreden	-22.384	96113.118	0.000	0.999
	Illnesses	0.002	0.00099	2.349	0.019*
	Hospitalizations	0.036	0.00636	5.696	0.000***
IFSAC category	Beef	-0.205	0.713	-0.288	0.774

	Chicken	0.026	0.490	0.053	0.958
	Crustaceans	-22.631	109582.258	0.000	0.999
	Dairy	-0.299	1.066	-0.281	0.779
	Eggs	-0.419	0.664	-0.632	0.528
	Fish	-23.166	78808.067	0.000	0.999
	Fruits	1.376	0.429	3.209	0.001**
	Herbs	-23.563	113564.758	0.000	0.999
	Multiple	-0.770	0.389	-1.980	0.048*
	Nuts-Seeds	1.191	0.882	1.350	0.177
	Other	-22.852	67135.122	0.000	0.999
	Other Meat	-23.076	113883.937	0.000	0.999
	Pork	0.258	0.622	0.415	0.678
	Root/Underground	-30.364	41844.157	-0.001	0.999
	Seeded Vegetables	-0.144	0.726	0.198	0.843
	Sprouts	0.684	0.821	0.834	0.405
	Turkey	-0.534	0.989	-0.540	0.589
	Vegetable Row Crops	-23.347	83248.176	0.000	0.999
	Null deviance:	552.50	2867 degrees of freedom		
	Residual deviance:	368.74	2812 degrees of freedom		
	AIC:	844.3			
	2 x log-likelihood:	-730.298			

Significance codes: 0 ‘***’ 0.001 ‘**’ 0.01 ‘*’ 0.05 ‘.’ 0.1 ‘ ’ 1.

Table S4. Result of causal mediation analysis with hospitalization and illness counts related to foodborne outbreaks caused by *Salmonella* serotypes as mediators.

Parameter*	Estimate	Standard error	95% CIL	95% CIU	P-value
R^{CDE}	0	0	0	0	0
R^{PNDE}	0	0	0	0	0
R^{TNDE}	0	0	0	0	0
R^{PNIE}	0.865	47.366	0.14	1.602	0.216
R^{TNIE}	0.865	47.255	0.163	1.6	0.216
R^{TE}	0	0	0	0	0
ER^{CDE}	-0.788	0.174	-0.95	-0.137	0
$ER^{INT_{ref}}$	-0.212	0.174	-0.863	-0.05	0.008
$ER^{INT_{med}}$	0.135	47.366	-0.602	0.86	0.216
ER^{PNIE}	-0.135	47.366	-0.86	0.602	0.216
$prop^{ER^{CDE}}$	0.788	0.174	0.137	0.95	0
$prop^{ER^{INT_{ref}}}$	0.212	0.174	0.05	0.863	0.008
$prop^{ER^{INT_{med}}}$	-0.135	47.366	-0.86	0.602	0.216
$prop^{ER^{PNIE}}$	0.135	47.366	-0.602	0.86	0.216
PM	0	0	0	0	0.216
INT	0.077	47.383	-0.05	0.814	0.208
PE	0.212	0.174	0.05	0.863	0.008

*R^{CDE}: *Controlled direct effect rate ratio;* R^{PNDE}: *Pure natural direct effect rate ratio;* R^{TNDE}: *Total natural direct effect rate ratio;* R^{PNIE}: *Pure natural indirect effect rate ratio;* R^{TNIE}: *Total natural indirect effect rate ratio;* R^{TE}: *Total effect rate ratio;* ER^{CDE}: *Excess relative rate due to controlled direct effect;* ER^{INTref}: *Excess relative rate due to reference interaction;* ER^{INTmed}: *Excess relative rate due to mediated interaction;* ER^{PNIE}: *Excess relative rate due to pure natural indirect effect;* ER^{CDE}*(prop): Proportion* ER^{CDE}; ER^{INTref}*(prop): Proportion* ER^{INTref}; ER^{INTmed}*(prop): Proportion* ER^{INTme}; ER^{PNIE}*(prop): Proportion* ER^{PNIE}*; PM: Overall proportion mediated; INT: Overall proportion attributable to interaction; PE: Overall proportion eliminated.*
